# A Metagenomic Advance for the Cloning and Characterization of a Cellulase from Red Rice Crop Residues

**DOI:** 10.3390/molecules21070831

**Published:** 2016-06-25

**Authors:** Carlos Meneses, Bruna Silva, Betsy Medeiros, Rodrigo Serrato, David Johnston-Monje

**Affiliations:** 1 Departament of Biology and Graduate Program in Agricultural Sciences, State University of Paraíba–UEPB, Campina Grande–PB 58429-500, Brazil; 2 Graduate Program in Agricultural Sciences, State University of Paraíba–UEPB, Campina Grande–PB 58429-500, Brazil; bruna-reggina@hotmail.com; 3 Departament of Biology, State University of Paraíba–UEPB, Campina Grande–PB 58429-500, Brazil; betsydantas@gmail.com; 4 Department of Biochemistry and Molecular Biology, Federal University of Paraná–UFPR, Curitiba–PR 81.531-980, Brazil; rvserrato@ufpr.br; 5 Indigo Agriculture, Boston, MA 02129, USA; damojomo@gmail.com

**Keywords:** plant compost, functional metagenomics, endoglucanase

## Abstract

Many naturally-occurring cellulolytic microorganisms are not readily cultivable, demanding a culture-independent approach in order to study their cellulolytic genes. Metagenomics involves the isolation of DNA from environmental sources and can be used to identify enzymes with biotechnological potential from uncultured microbes. In this study, a gene encoding an endoglucanase was cloned from red rice crop residues using a metagenomic strategy. The amino acid identity between this gene and its closest published counterparts is lower than 70%. The endoglucanase was named EglaRR01 and was biochemically characterized. This recombinant protein showed activity on carboxymethylcellulose, indicating that EglaRR01 is an endoactive lytic enzyme. The enzymatic activity was optimal at a pH of 6.8 and at a temperature of 30 °C. Ethanol production from this recombinant enzyme was also analyzed on EglaRR01 crop residues, and resulted in conversion of cellulose from red rice into simple sugars which were further fermented by *Saccharomyces cerevisiae* to produce ethanol after seven days. Ethanol yield in this study was approximately 8 g/L. The gene found herein shows strong potential for use in ethanol production from cellulosic biomass (second generation ethanol).

## 1. Introduction

As plant biomass is naturally recycled by microbes in the environment, a diverse spectrum of cellulolytic enzymes must exist with the ability to metabolize cellulose. Cellulose-degrading microbes produce endoglucanases [1], which are able to randomly cleave internal sites on crystalline surfaces of cellulose, generating new ends to the molecular chain. Cellobiohydrolases are then able to act in a progressive manner at the reducing end of the cellulose chain to release cellobiose as the main degradation product of cellulose.

Glycosyl hydrolases are classified into different families, based on the similarity of their protein sequences [2]. Cellulases and hemicellulases are known to be excellent biocatalysts and have a wide spectrum of industrial applications [3,4,5].

The biofuel industry is constantly searching for renewable alternatives to fossil fuels. Since there is so much cellulose in the world, development of an efficient means to break down this polymer into its constitutive sugars would allow production of very large amounts of ethanol using traditional fermentation based on the activity of yeasts. More specifically, cellulose must first be hydrolyzed by cellulases into cellobiose, which can then be converted by β-glucosidase to glucose, which can then feed into alcoholic fermentation. Extensive investigations have been carried out to find novel cellulases that can be applied to cellulosic biomass as a pretreatment to traditional fermentation; these studies usually focus on isolating, screening, and sequencing of genes from different organisms. Wastes from red rice agriculture and processing are one of the most abundant forms of cellulosic biomass in the northeast of Brazil, presenting an attractive target for production of cellulosic ethanol if a process for cellulose pretreatment could be developed. To find cellulosic enzymes capable of metabolizing the cellulose in red rice wastes, we proposed to isolate and characterize cellulases from organisms that are naturally able to convert red rice waste into glucose.

While metagenomic approaches have been used extensively to study extreme environments [6,7], less extreme environments that are favorable to life show a greater genetic diversity of microorganisms and are more likely to contain cellulase genes than environments where plant growth is rare or absent [6,7,8,9].

Bacteria and fungi are the main sources of cellulases for biotechnology [1], however, because the majority of the Earth’s microorganisms are not yet cultivable using traditional techniques [10], isolating genes from uncultured microorganisms is an attractive target for functional metagenomics [11]. This approach has already been used to clone and characterize cellulases [12]; however, there is still a large, untapped diversity of microbial cellulases in the many environments and species which have not been studied.

In this study, we apply functional metagenomics to discover a gene encoding a cellulolytic enzyme from composted red rice wastes. The process began with DNA extraction from composted red rice waste, the construction of a metagenomic library and the selection of clones capable of degrading carboxymethylcellulose (CMC) on agar plates. We identified an endoglucanase, EglaRR01, which has similarity to other cellulases from uncultivated bacteria. This enzyme was shown to be stable over a range of temperatures and pHs, and represents a significant advancement for the biotechnological degradation of cellulose for ethanol production.

## 2. Results

### 2.1. Construction and Screening of Metagenomic Libraries

**Figure 1:**
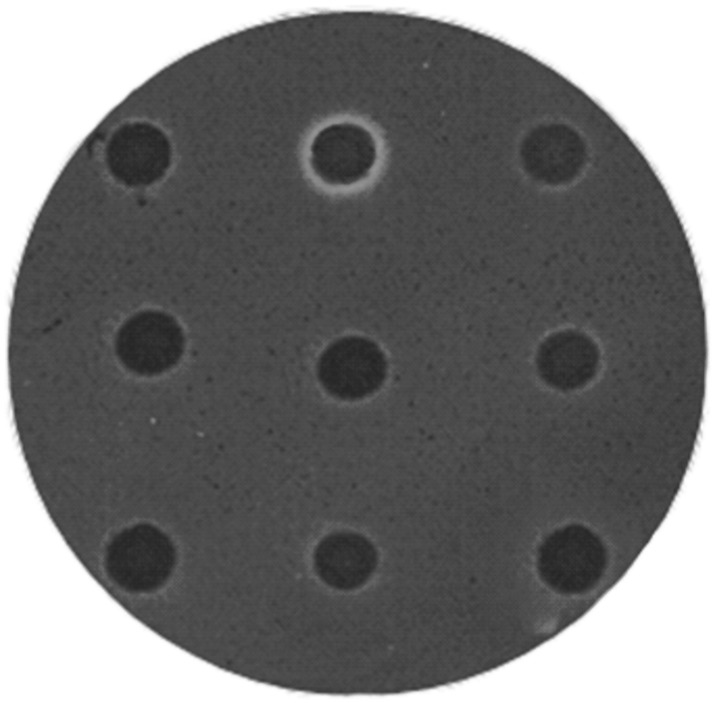
Cloning of a novel endoglucanase gene from red rice compost using a functional metagenomics approach. The CMC plate assay relies on the binding of Congo Red to cellulose [13].

A cosmid library of 10,000 clones was built using DNA isolated from a compost heap of red rice agricultural wastes; this library was called EglaRR. Restriction analysis with the *BamH*I enzyme of 50 randomly chosen clones showed that all cosmids contained DNA fragments with sizes from 15 to 60 kb. The average size of the inserted DNA library was approximately 40 kb. A clone with a carboxymethylcellulose-degrading phenotype (CMCase) was isolated after all EglaRR library clones were examined (Figure 1). This gene was named Endoglucanase 1 (EglaRR01) (deposited as KT779431).

### 2.2. Sequence Analyses of Cloned Cellulase Genes

**Figure 2:**
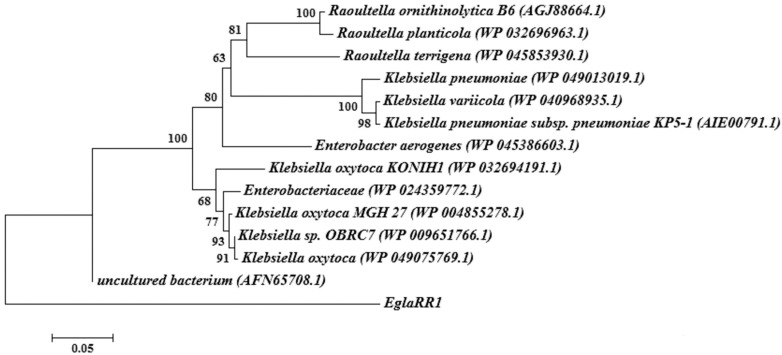
Classification of EglaRR01 by nucleotide and amino acid sequence analyses. Amino acid sequences of endoglucanases, including EglaRR01, were compared and analyzed phylogenetically using a neighbor-joining method. GenBank accession numbers are in parentheses. Phylogenetic analysis showed that EglaRR01 is closely related to cellulases from an uncultured species of *Enterobacter*.

The complete DNA sequence of the insert containing EglaRR01 was determined as 1278 bp. An alignment analysis by BLAST revealed the presence of an open reading frame (ORF) of 1080 bp, in the insert encoding a full-length gene for an endoglucanase (EglaRR01). The EglaRR01 gene was submitted to GenBank (KT779431) and encodes a predicted protein of 359 amino acids with a molecular mass of 40.1 kDa. The deduced amino acid chain of EglaRR01 was used for a BLAST against the NCBI and SwissProt databases. This search revealed that EglaRR01 belongs to the glycosyl hydrolase family 8 and that its amino acid identity is 69% with respect to an endoglucanase from an uncultured species of *Enterobacter* and 61% to the endo-1,4-d-glucanase of endonuclease III from *Klebsiella oxytoca*. A phylogenetic tree built from the amino acid sequences was constructed to verify the evolutionary relationship of the EglaRR01 to 13 known endoglucanases, including seven from *Klebsiella*. EglaRR01 is not closely related to any other members of the endoglucanase family, which suggests that EglaRR01 is a new type of endoglucanase (Figure 2).

### 2.3. Expression and Purification of the Recombinant EglaRR01

**Figure 3:**
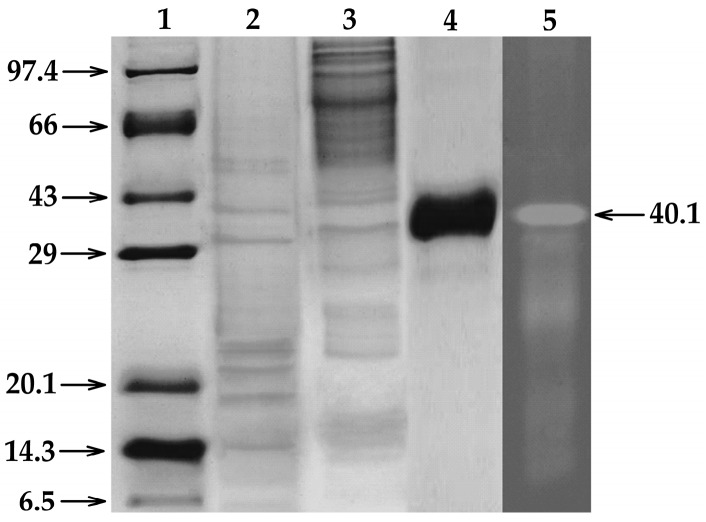
The recombinant EglaRR01 enzyme (0.1 μg) showed an active band at 40.1 kDa in the CMC zymogram. The three additional bands that appeared below 40.1 kDa in the CMC zymogram were probably caused by the action of partially degraded EglaRR01. **1**: Molecular weight marker (kDa); **2**: spin column portion of partly purified endoglucanase; **3**: ammonium sulfate (40%–60%) fraction; **4**: purified endoglucanase; and **5**: purified endoglucanase showing yellow opaque region in native gel.

In order to confirm the cellulolytic properties of EglaRR01, the recombinant protein was purified and in vitro tests were conducted using CMC zymograms to observe CMCase activity. The purified recombinant EglaRR01 protein (1 μg) was clearly active (Figure 3). Native-PAGE and SDS-PAGE gels were used for the qualitative characterization of cellulase activity. For Native-PAGE, the zymogram (0.1% CMC in the gel) showed a translucent yellow zone, indicating cellulolytic activity. For SDS-PAGE analysis, the enzyme approximate molecular weight was estimated to be 40.1 kDa.

The high specific activity of EglaRR01 on CMC and β-d-glucan (barley), and its limited activity on Avicel, cellobiose, and filter paper are consistent with the results found in the CMC zymogram, and with the phylogenetic analysis, which suggests that the recombinant protein is an endoglucanase. Interestingly EglaRR01 also showed highly specific activity against β-d-glucan (β-1→4 linked), like many endoglucanases that target the β-1→4 bond in CMC. However, EglaRR01 showed low laminarinase activity, not breaking β-1→3/1→6 linkages of laminarin as shown on Table 1.

### 2.4. Temperature and pH Influence on the Activity of EglaRR01

**Figure 4:**
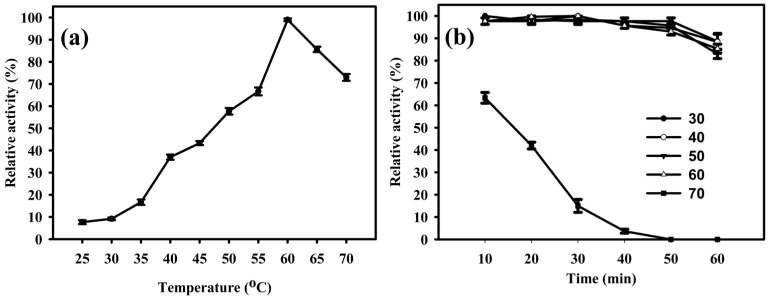
Effect of temperature on the activity and stability of EglaRR01. (**a**) Optimal temperature for EglaRR01 is 60 °C, as determined by measuring its enzymatic activity with 1% (*w/v*) CMC in 50 mM sodium acetate buffer, pH 5, at 25 to 70 °C in five degree increments; and (**b**) thermostability was determined by measuring the enzymatic activity of EglaRR01 after incubation at 30 to 70 °C in 10 degree increments for 60 min.

The EglaRR01 enzyme showed some activity at various temperatures between 25 and 70 °C, but had optimum activity when it was incubated at 60 °C (Figure 4a). Temperatures over 60 °C caused the enzyme to quickly lose its activity. When incubated for 1 h at different temperatures, EglaRR01 retained close to 90% of its activity, except at 30 °C when activity was drastically diminished over time (Figure 4b). Enzymatic properties of EglaRR01 at these lower temperatures were similar to the properties of other endoglucanases [14,15]. Cold-active enzymes are appealing due to their values in biotechnological applications. They are also useful tools for folding studies of proteins because of their high activity and stability at low temperatures [16]. Compost heaps are known to reach up to 70 °C, thus the optimum functioning temperature for EglaRR01 seems to be consistent with the environment from which it was isolated.

**Figure 5:**
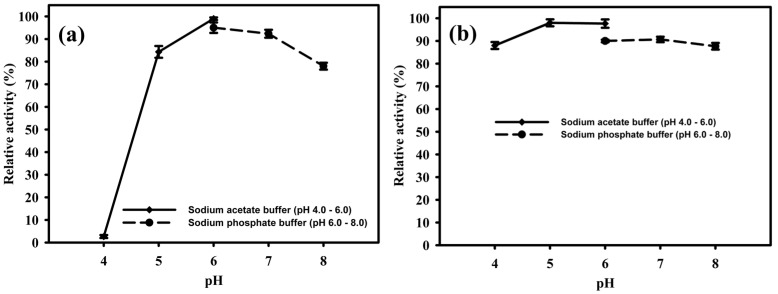
Effect of pH on the activity and stability of EglaRR01. (**a**) The optimal pH for EglaRR01 was determined by measuring the enzyme activity on 1% (*w/v*) CMC in 50 mM buffers at 65 °C with various pH values. The buffers used to establish the optimum pH and to assess pH stability were as follows: sodium acetate buffer (pH 4–6, ♦), and sodium phosphate buffer (pH 6–8, ●); and (**b**) to determine the pH stability of EglaRR01, the enzyme was incubated for 16 h at 4 °C in buffers of different pH values. The residual enzyme activity was measured under standard assay procedures. All measurements were carried out in triplicate.

The purified EglaRR01 enzyme was active at different pH ranges from 6.0 to 8.0. The optimal pH of the enzyme was 6.0, where it reached a maximum enzymatic activity in both acetate and phosphate buffers (Figure 5a). The enzymatic activity remained elevated when the pH was between 5.0 and 8.0. However, EglaRR01 activity was completely lost when the pH was reduced to 4.0. After 16 h incubation at 4 °C EglaRR01 still retained its activity at pH 4.0–8.0 (see Figure 5b). The pH range of the protein was comparable with that of alkaline cellulases reported elsewhere [17].

### 2.5. Production of Bioethanol from Red Rice Compost

The enzymatic extract of EglaRR01 was tested for its ability to degrade crop residues of red rice. Reducing sugars were detected at a concentration of 24 mg/g after enzymatic treatment of red rice crop residues, showing that this enzyme is able to convert cellulose containing agricultural wastes into glucose. The enzymatic pretreated red rice compost was then used as the sole carbon source to ferment with *Saccharomyces cerevisiae* BY4742 for seven days, which yielded 9.2 g/L of ethanol.

## 3. Discussion

Exploration of ‘‘metagenomes’’ for new enzymatic activities has been successful in a number of studies, which unequivocally demonstrate the potential of searching diverse environments for proteins with biotechnological potential [18]. In this work, a metagenomic library was built successfully using DNA extracted from crop residues of red rice, and functional screening allowed discovery of a novel cellulytic enzyme. We cloned and identified the novel endoglucanase, EglaRR01, which is closely related to the GH8 family of glycosyl hydrolases. Glycosyl hydrolases from family 8 (formerly known as D family cellulases) commonly break β-1→4 glycosidic linkages, being one of the first families to have its hydrophobic clusters characterized [19]. EglaRR01 showed an activity of 178 U/mg, which is approximately 72% higher than the GH8 family endoglucanase supplied by Megazyme (derived from *Aspergillus niger*, which has an activity of 86 U/mg [20]), and is stable on a wide range of pH and temperature. EglaRR01 shows much potential towards the efficient production of biofuels from lignocellulosic biomass.

The metagenomic approach employed in this study could also be employed for the isolation of endoglucanases (EC 3.2.1.4), which can release glucose from the ends of cellulose chains. The EglaRR01 library was cloned and hosted in *Escherichia coli*, which allowed functional characterization of transgenically-expressed enzymes and of the purified protein extracts. The molecular mass of an endoglucanase can vary over a wide size range. The smallest size of a reported endoglucanase is 6.3 kDa from *Cytophaga* [21], while other studied cellulases can reach up to 400 kDa as found on *Fusarium solani* [22]. The EglaRR01 protein purified in this study was about 40.1 kDa in mass, consistent with the size of proteins found in this group, which range from 35.9 to 659 kDa [23].

EglaRR01 was active over a wide pH range, maintaining 80% of its optimum activity at pH 8.0 and was stable between pH 4.0 to 8.0. The substrate degradation analysis indicated that EglaRR01 hydrolyzes β-(1→4) linkages more easily than β-(1→3) linkages. EglaRR01 showed activity against a wide variety of linear β-glucan derived from barley, but had a modest activity against mixed binding β-glucans derived from laminarin (6.5%). Microcrystalline cellulose derived from Avicel and filter paper were not degraded byEglaRR01 activity, whereas these substrates are promptly hydrolyzed by the action of other cellulases [24]. The high specificity of EglaRR01 to a given substrate is consistent with previous reports of cellulases belonging to that family [25].

The major restrictions of functional screening of metagenomic libraries is the need for a host to promote heterologous expression. The inability to recognize regulatory elements and the presence of different codon biases are some of the difficulties that limit success of functional metagenomics. Wang et al. [26] evaluated the overall phylogenetic distribution of CAZyme genes (carbohydrate-active enzymes) in rice straw compost enriched with manure, and found a microbial community involved in the process of lignocellulose decomposition. The CAZyme genes found were from Actinobacteria (46.1%), Proteobacteria (16.7%), Firmicutes (14.2%), Chloroflexi (7.7%), and Bacteroides (6.1%). Although the codons from the Shine-Dalgarno promoting regions of actinobacteria differ from those of *E. coli*, thus diminishing the probability of heterologous expression, the gene we have found in the culture remains of red rice that showed to be expressed in *E. coli*. We were one order of degree more efficient and only had to screen 10,000 clones to find one target gene. Our relatively high success rate could be, in part, explained by the wealth of cellulase gene diversity or the large Gram-negative bacterial population in red rice crop residues.

To facilitate the identification of cellulolytic activity in Petri dishes, a bacteriophage lambda expression system was used, facilitating the release of the cellulases expressed after lysis of *E. coli* host cells. Indeed, further efforts to purify cloned cellulases heterologously expressed in *E. coli* were successful despite residual cellulase activity that was detected in cellular extracts by more sensitive colorimetric assays.

The presented results suggest that, combined with other screening strategies including sequence-driven screening and high-throughput sequencing, functional mining of metagenomic libraries will not only capture new enzymes, but also provide insights into enzymatic hierarchy structure, as well as catalytic mechanisms in specific environmental niches.

The production of ethyl alcohol derived from lignocellulosic biomass comprises four processes: pre-treatment, enzymatic saccharification, fermentation, and ethyl recovery. The lignocellulosic residues have been examined by several studies involving microorganisms and enzymatic bioconversion with commercial cellulase [27,28,29,30,31]. Pre-treated plant cell wall polysaccharides are more susceptible to enzymatic hydrolysis, being broken into monomeric sugars which can then be fermented into ethanol [32]. We hope the enzyme we report discovering in this study contributes to the development of commercially viable cellulosic ethanol production.

## 4. Materials and Methods

### 4.1. Extraction and Purification of DNA from Environmental Samples

Crop residues of red rice (leaves, stems, spikelets, and straw in the proportion of 15:10:6:5 (wt %)) were collected and placed in composting cells at the State University of Paraíba, Campina Grande, Brazil, where they were incubated at temperatures ranging from 55 to 70 °C and at humidity ranging from 80% to 90%. For the extraction of DNA from these materials, we used a direct lysis method [33] with minor modifications.

Briefly, 1 g of plant sample was homogenized by vortexing in 2.6 mL of extraction buffer (100 mM Tris-HCl, pH 8.0; 100 mM sodium EDTA, pH 8.0; 100 mM sodium phosphate, pH 8.0; 1.5 M NaCl, 1% hexadecyltrimethylammonium bromide (CTAB)). Three freeze-thaw cycles were performed on the samples in liquid nitrogen and at 65 °C in a water bath. After adding 50 μL of proteinase K (20 mg/mL), samples were incubated at 37 °C for 30 min with continuous shaking at 120 rpm. Subsequently, 300 μL of 20% SDS (*w/v*) was added and the mixture incubated at 65 °C for 2 h with gentle shaking every 15–20 min. Supernatants were collected from the samples after centrifugation at 4000× *g* for 10 min and the resulting pellets were subjected to re-extraction in 2 mL of extraction buffer at 65 °C for 10 min. The combined supernatants were then mixed with 1/10 volume of 10% CTAB (*w/v*) [34] and centrifuged again. The resulting supernatant solutions were extracted with isoamylic chloroform–alcohol (24:1 *v/v*) and the DNA precipitated with isopropanol, washed with 70% (*v/v*) ethanol, dried, and resuspended in 100 of 10 mM Tris HCl, pH 8.5. For DNA purification, the crude DNA extracts were purified by size exclusion chromatography columns CHROMA SPIN + TE-1000 (BD Biosciences Clontech, Heidelberg, Baden-Württemberg, Germany), equilibrated in 10 mM Tris-HCl, pH 8.5, according to the manufacturer’s recommendations.

### 4.2. Construction of Metagenomic DNA Library and Screening

A metagenomic library was constructed using the pWEB::TNC Cosmid Cloning Kit (Epicentre, Madison, WI, USA) according to the manufacturer’s instructions. The purified DNA had its ends repaired with T4 DNA polymerase and T4 polynucleotide kinase to generate blunt ends. DNA with repaired ends were separated on agarose gel. The DNA fragments between 40 and 50 kb were recovered from the gel and ligated to a vector cosmid pWEB::TNC which was linearized at the unique site *Sma*I and dephosphorylated. The ligated products were transformed into *E. coli* EPI100 cells infected. The library colonies were screened for endo-1.4-β-glucanase (carboxymethylcellulase) activity according to protocols described by Teather and Wood [13]. The clone that tested positive for endo-1.4-β-glucanase activity (EglaRRN01) was sequenced.

### 4.3. Sequence and Phylogenetic Analyses

Potential open reading frames in the cloned sequence were identified using ORF finder at the National Center for Biotechnology Information (NCBI). Sequences closely related to candidate cellulases were recognized with the BLASTP and BLASTN algorithms in NCBI. For phylogenetic analysis sequences of the cloned cellulase, its highest Genbank matches, and some sequences of representative cellulases from different families of glycosyl hydrolases were selected. The phylogenetic tree was generated with ClustalW 1.81 (Heidelberg, Baden-Württemberg, Germany) and MEGA 6.0 (Tempe, AZ, USA) using the neighbor-joining technique.

### 4.4. Expression and Purification of a Recombinant Endoglucanase

Having identified EglaRRN01 as a cellulase, we intended to subclone it using PCR, while excluding the N-terminal signal peptide. This gene was amplified by polymerase chain reaction (PCR) using pEglaRRN01 as template and the following primers: sense primer 5′-ATAGCATGCCCGCTCGTGCGAAGATCC-3′ (containing the *Sph*I site at the end 50) and an antisense primer 5′-ATAGTCGACCATGTCCCGCAATGCCGC-3′ (containing a *Sal*I site at the end 50). The amplified DNA was digested with *Sal*I and *Sph*I (Promega, Madison, WI, USA) before ligation to the pQE31 vector digested with the same enzymes (Qiagen, Valencia, CA, USA) resulting in plasmid pEglaRR01. The recombinant plasmid pEglaRR01 was then used to transform *E. coli* M15 (Qiagen). His-tagged EglaRR01 was expressed and purified using nickel-nitrilotriacetic acid (Ni-NTA) agarose resin (Qiagen) according to the manufacturer’s instructions.

### 4.5. Endoglucanase Activity Assay

The enzyme’s ability to degrade CMC was determined by measuring the concentration of reducing sugars using the dinitrosalicylic acid method (DNS) [35]. The reaction mixture contained 0.5 mL of 1% CMC (*w/v*) in 50 mM sodium phosphate buffer, pH 7.0, and 0.5 mL of enzyme solution, and was incubated at 65 °C for 20 min. The amount of reducing sugars produced was determined after the incubation. One enzymatic unit corresponds to the equivalent of 1 μmol of glucose produced per minute. Protein concentrations was determined by the Bradford method [36] using an assay kit for the quantification of protein (Bio-Rad, Hercules, CA, USA). A standard curve of protein concentration was created using known concentrations of bovine serum albumin.

### 4.6. CMC-Zymogram Analysis

Sodium dodecyl sulfate (SDS) polyacrylamide gel electrophoresis (SDS-PAGE) [37] was performed with some modifications to detect cellulase and β-glucosidase activity using zymogram [38,39]. For the CMC zymogram, an SDS-PAGE 12% gel was prepared containing 0.1% carboxymethylcellulose (Sigma-Aldrich, St. Louis, MO, USA). The enzyme extracts were put into half of the gel at a concentration of 40 μg protein per line. This pattern was repeated to entrain the other half of the gel so that it could be cut vertically in half after electrophoresis to produce two identical acrylamide gels. The first half of the gel was stained with colloidal blue stain for visualization of proteins. The second half was used for zymogram analysis. For zymogram analysis, the gel was washed in sodium citrate buffer (50 mmol/L, pH 5.5) containing 1% Triton X-100 for 1 h at room temperature to remove SDS [38]. This was followed by incubation for 1.5 h in sodium citrate buffer (50 mmol/L, pH 5.5) to allow for enzymatic activity to the substrate. Then, the gel was stained with Congo Red (Sigma-Aldrich) at 0.1% for 30 min and destained in 1 mol/L NaCl to reveal the lightening zones. The gel was imaged under ultraviolet light and aligned with colloidal blue stained gels.

### 4.7. Biochemical Characterization of Endoglucanase EglaRR01

The optimum temperature for EglaRR01 activity was determined by measuring the activity of a control endoglucanase on CMC 1% (*w/v*) (in 50 mM sodium acetate buffer, pH 5.0) from 25 to 70 °C with differences in five degree increments. The thermal stability was examined by incubating the enzyme at 25 to 60 °C in increments of 10 degrees for 60 min. The residual enzyme activity was, again, checked using 1% (*w/v*) CMC in 50 mM sodium acetate buffer, pH 6.0, at 65 °C [40].

The optimum pH for enzymatic activity of EglaRR01 was also determined by measuring its enzyme activity in CMC 1% (*w/v*) in 50 mM buffer at pH values ranging from 4.0 to 8.0, at 65 °C. Sodium acetate buffer was used at a pH between 4.0 and 6.0. Sodium phosphate buffer was used for pH ranges between 6.0 and 8.0. To determine the pH stability of the enzyme, the extract containing the enzyme was incubated at different pHs as mentioned above, followed by storage at 4 °C for 16 h. The residual enzyme activity was measured under standard assay procedure (1% CMC in 50 mM sodium acetate buffer, pH 6.0, at 65 °C) [40].

To investigate the substrate specificity, enzymatic activities were tested under optimal conditions for 20 min with 1% (*w*/*v*) of the following polysaccharide substrates: Avicel (Fluka, Sigma-Aldrich), CMC (Sigma-Aldrich), cellobiose (Sigma-Aldrich), cellulose fiber (Sigma-Aldrich), β-d-glucan (Barley, Sigma-Aldrich), filter paper (Whatman No. 1, Maidstone, Kent, UK), laminarin (Sigma-Aldrich), lichenan (Sigma-Aldrich) and xylan-brichwood (Sigma-Aldrich). 4-Nitrophenyl β-d-cellobioside (pNPC) (Sigma-Aldrich), the final concentration was 1 mM [40].

### 4.8. Saccharification of Red Rice Residues by Endoglucanase EglaRR01

The enzymatic extract EglaRR01 was tested for its ability to degrade red rice residues. The residues were ground and separated on 40 mesh sieves. The enzyme activity in degrading red rice remains was determined by measuring the concentration of reducing sugars using the dinitrosalicylic acid method (DNS) [35]. Briefly, the reaction mixture that contained 0.5 mL of red rice residues 1% (*w/v*) in 50 mM sodium acetate buffer, pH 7.0, and 0.5 mL of enzyme solution, was incubated at 60 °C for 20 min. Subsequently, the amount of reducing sugars produced were determined. One enzymatic unit corresponds to the equivalent of 1 μmol of glucose produced per min. Protein concentrations were determined using the Bradford method [36] using an assay kit for the quantification of protein from Bio-Rad. A standard curve of protein concentration was created using known concentrations of bovine serum albumin (Sigma-Aldrich).

### 4.9. Bioconversion of Rice Straw Residues into Bioethanol Using Endoglucanase EglaRR01

#### 4.9.1. Enzymatic Saccharification

One gram of red rice residue was pre-treated with 0.5 U/g of purified enzyme extract in 50 mM sodium acetate buffer, and then the mixture was packed in 100 mL Erlenmeyer flasks with a magnetic stir bar. The final volume was adjusted to 50 mL using 50 mM sodium acetate buffer. The Erlenmeyer flasks were incubated at 60 °C for 12 h with stirring. After incubation, the reaction was completed by adding 3 mL of DNS 1% for each 1 mL of the reaction mixture, which was subsequently heated for 10 min. The amount of reducing sugars in the reaction was measured as described above.

#### 4.9.2. Detoxification

The pre-treated samples, and purified enzyme extract were mixed with activated charcoal (20:1 *w/w*) and then stirred for 2 days at ambient temperature with a magnetic stirrer. The samples were filtered through filter paper No. 5 (Whatman), after treatment with charcoal [31]. The filtrate was subjected to measurement of reducing sugars as described above.

#### 4.9.3. Fermentation

The ethanol fermentation was conducted with the yeast *S. cerevisiae* BY4742 grown in YEPD medium. A loopful of yeast was inoculated and incubated into a liquid fermentation broth at 30 °C at 200 rpm for 24 h. The count of yeast in the fermentation sample was 9 × 10^7^ CFU/mL.

The medium used in the fermentation for ethanol production was composed of: glucose (from the sugar solution obtained from the saccharified crop residues of red rice), KH_2_PO_4_ 0.1%; (NH_4_)_2_SO_4_ 0.5%; MgSO_4_·7H_2_O 0.05%; and yeast extract 0.1%, wherein the pH of the medium was adjusted to pH 5.0. The medium was introduced into Erlenmeyer flasks of 250 mL containing 100 mL of the fermented solution. The pH of the medium adjusted to 5.0 and then inoculated with 10% saccharified solution from the pre-treated sample of red rice residues. All repetitions were incubated at 30 °C at 200 rpm agitation for seven days. The ethanol content was measured seven days after the fermentation process began [28,41].

#### 4.9.4. Ethanol Estimation

One mL of the fermented filtrate was introduced into the distillation flask of 500 mL of capacity containing 30 mL of distilled water. Distilled samples were collected in a 50 mL flask containing 25 mL of a potassium dichromate solution (33.76 g K_2_Cr_2_O_7_ dissolved in 400 mL distilled water with 325 mL of sulfuric acid in the final volume of 1 L). About 20 mL of sample was collected and kept in a water bath at 62.5 °C for 20 min.

The flasks were cooled to room temperature and the volume was topped up to 50 mL. Five mL of this solution was diluted with 5 mL of distilled water to measure the optical density of the solution at 600 nm using a spectrophotometer [42]. A standard curve was performed under the same conditions using standard ethanol solution containing 2% to 14% (*v/v*) ethanol in distilled water and then the ethanol content of each sample was estimated [31].

## 5. Conclusions

In conclusion, an enzyme belonging to family 8 of glycosyl hydrolases was isolated successfully using a functional approach to screen a metagenomic library built from DNA isolated from red rice crop residues. The screen involved looking for cellulolytic activity in a heterologous expression in *E. coli*. EglaRR01, and subsequent purification and characterization of the recombinant protein. The enzyme exhibited thermal stability and efficiency in a wide pH range, with a significant degradation speed for a variety of beta-glucans: β-(1→4) and β-(1→3/1→4). These characteristics make EglaRR01 a strong candidate suitable for future industrial use.

## Figures and Tables

**Table 1:** Substrate specificity of EglaRR01.

Substrate	Specific Activity (U/mg)
β-d-glucan (Barley)	1487.9
CMC	1002.5
Avicel	0
Cellulose fiber	5.8
Cellobiose	0
Xylan-birchwood	500.4
Lichenan	433.9
Laminarin	6.5
Filter paper	2.9

EglaRR01 shows highly specific activity towards CMC, but limited activity towards Avicel, filter paper and Cellobiose. Surprisingly, EglaRR01 demonstrated highly specific activities toward natural substrates, such as β-d-glucan and lichenan (containing β-1→4/1→3 linkages), compared to that against artificial CMC (β-1→4 linkage). Given that laminarinase activity (β-1→3/1→6 cleavage) of EglaRR01 was low, the difference in activity towards multiple substrates is not due to β-1→3 cleavage potential. One unit is defined as the equivalent of 1 μmol of glucose produced per minute.

## Abbreviations

The following abbreviations are used in this manuscript:

CMC: carboxymethylcellulose
pNPC: 4-nitrophenyl-β-d-cellobioside
CBM: carbohydrate binding motif
Ni-NTA: nickel-nitrilotriacetic acid
DNS: dinitrosalicylic acid
CFU: colony-forming unit
